# Dual-Tracer Autoradiography and Positron Emission Tomography (PET) Scans Using In-Yolk-Sac Tracer Delivery in the Chicken Chorioallantoic Membrane (CAM) Tumor Model

**DOI:** 10.3390/biomedicines14071515

**Published:** 2026-07-06

**Authors:** Emil L. Villumsen, Signe Bauenmand, Marie B. Thuesen, Mikkel H. Vendelbo, Lars Thrane, Jörg Männer, Niels Bassler, Michael R. Horsman, Michael Pedersen, Morten Busk

**Affiliations:** 1 Comparative Medicine Lab, Department of Clinical Medicine, Aarhus University, 8200 Aarhus, Denmark; mathu@clin.au.dk (M.B.T.); lath@clin.au.dk (L.T.); michael@clin.au.dk (M.P.); 2 Department of Experimental Clinical Oncology, Aarhus University Hospital, 8200 Aarhus, Denmark; mike@oncology.au.dk (M.R.H.); morten@oncology.au.dk (M.B.); 3 Molecular Bone Histology Team, Department of Forensic Medicine, Aarhus University Hospital, 8200 Aarhus, Denmark; au635002@forens.au.dk; 4 Department of Nuclear Medicine & PET Centre, Aarhus University Hospital, 8200 Aarhus, Denmark; mhve@biomed.au.dk; 5 Department of Biomedicine, Aarhus University, 8000 Aarhus, Denmark; 6 Institute for Anatomy and Cell-Biology, UMG, University of Göttingen, 37075 Göttingen, Germany; jmaenne@gwdg.de; 7 Danish Centre for Particle Therapy, Department of Clinical Medicine, Aarhus University Hospital, 8200 Aarhus, Denmark; bassler@clin.au.dk; 8 Department of Clinical Medicine, Aarhus University, 8200 Aarhus, Denmark

**Keywords:** tumor metabolism, tumor microenvironment, chorioallantoic membrane, autoradiography, positron emission tomography, nuclear medicine

## Abstract

**Background**: Routine use of the chorioallantoic membrane (CAM) tumor model in nuclear imaging studies is hampered by small tumors, embryonic movements and laborious volume-restricted intravenous tracer/drug administration. We sought a workaround by using fast-growing tumors, high-resolution autoradiography and non-intravenous tracer administration. **Methods**: Dekalb White chicken eggs were grafted with C3H mammary carcinoma fragments or MOC2 oral squamous cell carcinoma fragments from donor mice. The tumor uptake of ^18^F-fluorodeoxyglucose (FDG) following in-yolk-sac injection, dripping after CAM scoring or allantoic cavity injection was evaluated using positron emission tomography (PET) and autoradiography. Using in-yolk-sac injection, eggs were administered different tracer mixtures, namely (1) pimonidazole (hypoxia-marker), FDG and ^14^C-2-deoxyglucose (^14^C-2DG), (2) pimonidazole, FDG and ^14^C-acetate or (3) pimonidazole, the hypoxia-selective tracer ^18^F-fluoroazomycin-arabinoside (FAZA) and ^14^C-2DG. For comparison, tumor-bearing mice were administered FDG/^14^C-acetate/pimonidazole. Gross tumor uptake was evaluated using PET. Tumor cryosections were analyzed using dual-tracer autoradiography. Complementary autoradiograms were co-registered, covered by a square grid (0.5 × 0.5 mm). Pearson correlation coefficients (PCC) were calculated from scatterplots. **Results**: C3H tumors reached a mean weight (with 95% confidence interval) of 0.32 g (0.28–0.37 g), while for MOC2, it was 0.19 g (0.09–0.29 g). In-yolk-sac tracer injection was simple and effective, producing high tracer uptake and contrast 3 h post-administration. Spatial tracer overlap (PCC) was: FDG vs. ^14^C-2DG, 0.95–0.97; FAZA vs. ^14^C-2DG, 0.71–0.79 and FDG vs. ^14^C-acetate, 0.26–0.84 (0.15–0.76 in mice). Pimonidazole revealed tumor hypoxia. **Conclusions**: Direct-grafting from donor mice generated larger tumors than previously reported. In-yolk-sac tracer administration was practical and allowed larger injected volumes. Autoradiography revealed that: (1) FDG and ^14^C-2DG can be used interchangeably, (2) ^14^C-2DG was elevated in FAZA-positive areas, suggesting that in some tumors FDG-PET may provide information on the intratumoral distribution of hypoxic areas, and (3) FDG and ^14^C-acetate showed variable overlap. We conclude that in-yolk-sac tracer injection and autoradiography simplify and optimize CAM-based nuclear imaging research.

## 1. Introduction

**Figure 1:**
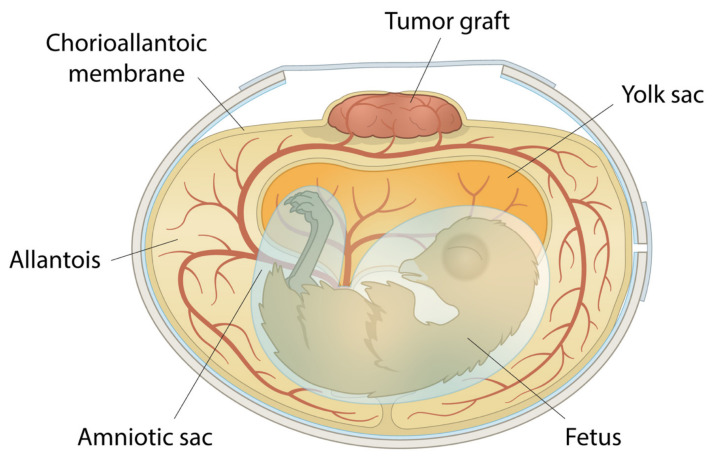
Fundamental CAM model morphology of a ~14-day old embryo with a developing tumor graft and important embryological structures, modified from [4].

The use of effective preclinical in vivo models, typically tumor-bearing mice, in oncological nuclear medicine research is paramount to ensure continuous development of novel experimental positron emission tomography (PET) tracers and to define their relationship with well-established tracers and underlying tumor biology. The chorioallantoic membrane (CAM) model of the domestic chicken (*Gallus gallus domesticus*) offers several advantages over traditional murine models, including lower cost, fewer ethical concerns, simplified handling and reduced regulatory requirements [1,2,3]. An example of a tumor-bearing CAM model on embryonic development day (EDD) 14 is illustrated in Figure 1, with the important structures highlighted.

Although PET scans have previously been employed [5,6,7], the use of the CAM model in nuclear medicine experiments has been limited. PET is constrained by low spatial resolution and movement artifacts, and this is particularly problematic in the CAM model, where tumors can only be grown for a maximum of ~10–12 days, with the tumor being embedded in a membrane that overlays an increasingly lively fetus. High-resolution autoradiography presents a compelling, yet highly underutilized, alternative in CAM studies. Specifically, autoradiography (1) improves image resolution substantially from 1 to 2 mm in small-animal scanners to ~100 µm; (2) allows a direct comparison of two differently labeled (e.g., ^14^C and ^18^F) tracers in a single tissue section, thus avoiding co-registration inaccuracies and movement artifacts; and (3) allows regional tracer retention to be directly correlated with underlying tumor histology, microenvironment (e.g., oxygenation), and biology, including the expression of various macromolecules. Hypoxia is widespread in solid tumors, and post-treatment survival of treatment-resistant hypoxic cells is a leading cause of relapse and poor prognosis [8]. Hypoxia-selective PET tracers, like ^18^F-Fluoroazomycin-arabinoside (FAZA), may guide treatment intensification to overcome resistance, but hypoxia-PET has never been implemented in routine clinical use. Since hypoxia stimulates glucose use (anaerobic glycolysis), ^18^F-fluorodeoxyglucose (FDG) and FAZA may provide overlapping information, but the results have been inconsistent, possibly due to the complexity of clinical multi-tracer trials [8]. In addition, recent studies have demonstrated that acetate may serve as an important source of acetyl-CoA used for lipid synthesis [9]. Of note, acetate was shown to be a quantitatively important epigenetic metabolite that promotes lipid synthesis in hypoxic and/or nutrient-deprived cells in vitro [10,11]. Targeting of acetate transmembrane uptake (e.g., monocarboxylate transporters) [12] or metabolism (e.g., acetyl-CoA synthetase) [13] may thus have anti-tumor effects, possibly due to killing of treatment-resistant hypoxic acetate-dependent cells [14]. Accordingly, ^11^C-acetate-PET may become valuable for treatment planning and treatment-efficacy monitoring. However, the intratumoral distribution of acetate retention and its spatial relationship to FDG-PET is unknown. Based on the limitations inherent to the CAM model, we sought to improve the user-friendliness of the CAM model and provide new areas of application, including unraveling of the spatial relationship between established and emerging tracers. Specifically, our aims were to investigate whether the following statements are correct: (1) use of fresh tumor fragments obtained from fast-growing murine tumors allows us to establish larger tumors than reported previously, (2) straightforward non-IV tracer administration is a feasible alternative to intravenous injections, (3) dual-tracer autoradiography can circumvent inherent problems of small tumors and movement artifacts when using the CAM model and (4) hypoxia-PET (e.g., FAZA) and ^14^C-acetate-PET provide similar or complementary information regarding target characterization compared to FDG-PET.

## 2. Materials and Methods

### 2.1. CAM Model Creation

Commercial Dekalb White chicken eggs were marked with an “X” on the uppermost part of the shell when turned sideways, then rested for 24 h at 17 ± 1 °C; they were subsequently incubated in a HEKA-Laboratory-Incubator “In-Ovo” (HEKA Brutgeräte GmbH & Co. KG, Rietberg, Germany) sideways at 37.8 °C and 67% relative humidity, with 12 automatic turns per day and the “X” facing upwards. On EDD 3, the egg turning was discontinued, and eggs were transferred in batches of five to a laminar-flow hood. A Dremel model 4250 rotary tool (Dremel, Bosch Power Tools B.V., Breda, The Netherlands) with a silicon carbide grinding stone attachment was used to create a small blunt-end indentation to aspirate 2 mL of albumen using a sterile 23 G needle, carefully avoiding the yolk. A guiding groove corresponding to the desired window size was drilled around the “X.” Shell fragments and underlying shell membranes were carefully removed using tweezers to expose the fetus, and the embryo viability was confirmed by direct visualization of the fetus, its vasculature, and its heartbeat. Unfertilized or nonviable eggs were discarded. The shell windows were sealed with transparent film dressing, and the eggs were returned to the incubator under identical conditions until grafting on EDD 8. Embryo viability was monitored using a candling lamp, and nonviable embryos were removed. At the end of each experiment, embryos were euthanized by decapitation.

### 2.2. Tumor Harvest and Grafting

A total of 14 tumor-bearing mice were used for the experiments, including initial tests of the CAM model, autoradiography tests, and the specific experiments presented in the manuscript. We used 13 CDF1 mice, 8 bearing C3H mammary carcinoma flank tumors and 5 bearing C3H mamary carcinoma subcuteaneous (SC) foot tumors. 1 C57BL/6J mouse bearing a MOC2 oral squamous cell carcinoma flank tumor was used. Mice were euthanized by cervical dislocation. Tumors for grafting were excised immediately after euthanasia under sterile conditions and cut to grafting size in a laminar-flow hood, resulting in 20–25 grafts from each tumor, with the mean weight and 95% confidence interval (CI) of 0.049 g (0.046–0.052 g) for the C3H and 0.038 g (0.034–0.042) for MOC2. When grafting, groups of five eggs were reopened in a laminar-flow hood by removing the transparent film dressing. A small area of the CAM was scored in each egg with a sterile 23 G needle protector, and a graft was placed on the membrane using tweezers. The shell windows were resealed, and the eggs were returned to the incubator under usual conditions until the experiments were performed. Graft weight was determined by weighing 1.5 mL Eppendorf tubes containing the graft and 100 µL sterile NaCl (9 mg/mL) before and after implanting the graft. One group (*n* = 21) was incubated until EDD 17 before experiments and the other (*n* = 34) until EDD 18. This was due to limitations on how many tumors could be analyzed per day. Larger tumors were favored at EDD 17 to ensure a relevant tumor size for all experiments.

### 2.3. Non-IV Administration Methods

For allantois injection, a 23 G needle was used to deliver compounds directly into the allantois under direct visual guidance. Dripping after scoring involved first scoring the CAM as in the grafting procedure, well away from the tumor, and then delivering the tracer bolus onto that scored portion of the CAM. In-yolk-sac bolus injections were performed by inserting a 23 G syringe into the yolk sac by hand under direct visual guidance. Volumes administered ranged from 0.2 mL to 0.75 mL due to the decay of ^18^F-based tracers throughout the experimental days.

### 2.4. Statistics

Statistical analyses were performed with all two-sided tests, and *p* < 0.05 was considered statistically significant. Embryo survival from EDD 0 to 18 was estimated by Kaplan–Meier analysis. Survival rate at EDD 18 of the CAM models and the 95% CI was obtained from the Kaplan–Meier analysis. Eggs sacrificed for autoradiography experiments at EDD 17 were censored at that time. Tumor weight at harvest was treated as a continuous variable and normality was assessed by QQ-plots. When tumor weight deviated from normality, it was log-transformed and re-checked on QQ plots. All statistical summaries of tumor weight were calculated on the log scale when relevant and back transformed for reporting. The tumor take-rate was defined as >100% increase in tumor weight from EDD 8 to EDD 18. The 95% CIs were calculated for each tumor type and for each administration method. Statistical analyses were performed in Stata 18 SE (StataCorp, College Station, TX, USA).

### 2.5. Radioactive Tracers and Pimonidazole

CAM models received ~35 MBq of PET tracers FDG or FAZA (half-life: 110 min) alone (PET/single-tracer autoradiography) or in relevant combinations with the long-lived (half-life: 5700 years) PET tracer analogs ^14^C-acetate (100 kBq) or 2-deoxy-D-[1-14C]glucose (^14^C-2DG) (37 kBq) for dual-tracer autoradiography. In each CAM model exposed to pimonidazole, 0.25 mL of 200 mg/mL pimonidazole in saline was administered for a total of 50 mg of pimonidazole per CAM model. In multi-tracer experiments, all tracers (including pimonidazole) were mixed and administered as a single bolus.

FDG and FAZA were produced locally at the Department of Nuclear Medicine and PET Centre, Aarhus University Hospital. ^14^C-labeled tracers were obtained from American Radiolabeled Chemicals (Saint Louis, MO, USA). Pimonidazole was a gift from J.A. Raleigh (University of North Carolina, Chapel Hill, NC, USA).

### 2.6. PET Scans

PET and MRI scans were performed with a dedicated small-animal 1 Tesla PET/MRI system (Mediso Medical Imaging Systems, Budapest, Hungary). For dynamic PET, tracer administration was immediately followed by placement of the eggs into the scanner, and imaging was conducted continuously over a 3 h uptake period, with a heating pad adjusted to 37.8 °C underneath the eggs. Static PET scans were performed after a 3 h uptake period in a temperature-controlled environment (~37 °C). To quantify tracer uptake, we manually delineated a volume of interest around the tumor using InterView Fusion software version 3.11.005.0000 (Mediso, Budapest, Hungary).

### 2.7. Dual-Tracer Autoradiography

After tracer administration and a 3 h uptake period at ~37 °C, tumors were excised and frozen in pre-cooled (−40 °C) iso-pentane for subsequent embedding in Tissue Tek O.C.T. and cryosectioning. Neighboring sections (10 µm) were cut from different tissue depths to ensure a selection of sections without cutting artifacts representing various tumor parts. Cryosections were then exposed to BAS-SR 2025 phosphor imaging plates (Fuji Photo Film Co., Ltd., Tokyo, Japan) immediately after sectioning for ∼1.5 h. Plates were analyzed for the distribution of activity at a resolution of 25 µm using an Amersham Typhoon Biomolecular Imager (GE healthcare, Marlborough, MA, USA). To capture the long-lived tracers (^14^C-2DG or ^14^C-acetate), exposure to the phosphor plates was repeated after a minimum of 36 h to allow the complete decay of ^18^F-based tracers (FDG or FAZA). The second exposure lasted 5–6 days to compensate for the low radioactivity of the ^14^C tracers. ^14^C exposures were conducted in a freezer maintained at −20 °C, to lower tissue/epitope degradation, while using lead plate shielding to minimize background radiation contamination.

### 2.8. Autoradiogram Tracer Distribution Analyses

Autoradiograms were visualized and analyzed using the Multi Gauge V3.0 software (Fujifilm, Tokyo, Japan). The extent of tracer distribution similarity between autoradiograms was quantified by covering complementary autoradiograms with a square grid with a cell size of 0.5 × 0.5 mm, which was chosen as a compromise that ensures a grid-cell size well above the true plate resolution (~100 µm) while still being able to capture intratumoral heterogeneity. The resulting scatterplots were used to quantify tracer distribution similarities (Pearson regression coefficients). Furthermore, the dynamic range for the two glucose tracers FDG and ^14^C-2DG was compared by calculating the ratio between the average signal of the 10 most and 10 least intense voxels in corresponding autoradiograms. Raw data (PSL/mm^2^) were used for regression analyses and dynamic range calculations, since meticulous correction for differences in injected doses, weight of eggs, plate exposure details (initiation and duration), and inclusion of appropriate standards for ^18^F and ^14^C do not affect the results. For accurate calculation of the dynamic range, signal intensity was corrected for background signal, defined in an area with no tissue sections.

### 2.9. Pimonidazole Analysis

Selected tissue sections were thawed and fixed in formalin. Endogenous peroxidases were blocked with H_2_O_2_ and rinsed in phosphate-buffered saline. Subsequent antibody staining steps were performed using a LabVision Autostainer 480 (LabVision, Fremont, CA, USA). Sections were incubated for 40 min with an antibody specific to pimonidazole (Pab2627 from Hypoxyprobe, Burlington, MA, USA) followed by rinsing. Finally, sections were incubated with anti-rabbit IgG-horseradish peroxidase-conjugated polymers with a 30 min incubation time (EnVision rabbit reagent, K4003; DakoCytomation, Glostrup, Denmark). Stained sections were then counterstained with Mayer’s hematoxylin, rinsed with water, and fixed in dibutylphthalate polystyrene xylene. Pimonidazole-stained sections were digitized using a Hamamatsu Tissue Scanner 2.0 HT slide scanner (Hamamatsu Photonics, Hamamatsu City, Japan).

### 2.10. In Vivo Mouse Experiment

Five CDF1 mice were inoculated SCin the foot pad of the right hind leg with a C3H mammary carcinoma from a donor mouse. When ready for experiments, mice were injected intraperitoneally with a bolus of FDG (~20 MBq), ^14^C-acetate (200 kBq) and pimonidazole (60 mg/kg) [15]. Tumors were harvested 1 h post-tracer administration and tumor cryosections were prepared and analyzed using dual-tracer autoradiography and pimonidazole staining, as described for CAM-grown tumors. No animals were excluded.

## 3. Results

### 3.1. Tumor Sizes and Survivability of the Model

A total of 119 fertilized eggs were used for establishing the model, including initial testing and all experiments. Of these, 55 were specifically used for autoradiography experiments. Our model had a total survival rate of 79%, 95% CI (70.21–85.14) to EDD 18, showing a survivability akin to the natural hatchability of older hens (73%) but lower than the maximum natural hatchability of ∼96% [16]. The C3H mammary carcinomas analyzed (*n* = 68) grew to a mean weight of 0.32 g (0.28–0.37 g) with a take rate of 81%, 95% CI (70–89%). To further validate the general applicability, we established MOC2 oral squamous cell carcinomas (*n* = 4) which grew to 0.19 g, 95% CI (0.09–0.29 g), with a take rate of 67%, 95% CI (30.0–90.3%) (see Supplementary Material Table S1). Assuming a tissue density of 1 g/cm^3^ [17,18], C3H mammary carcinoma weights corresponded to a mean volume of ~320 mm^3^ and the MOC2 oral squamous cell carcinoma weights to a mean volume of ~190 mm^3^.

### 3.2. Suitability of Different Tracer Administration Routes

Tracer administration was attempted in 55 tumor-bearing CAM models. Success of administration was evaluated from an autoradiographic visualization of the tracer uptake, which was typically binary in nature (strong or very weak/absent signal). Direct dripping without scoring the CAM (see Supplementary Figure S1) was unsuccessful (*n* = 1), and in one egg, tumor tracer retention could not be confirmed since the tumor was too small for cryosectioning. Success rates of tracer deliveries in 53 eggs were: yolk sac injection 91% (*n* = 34), dripping after scoring 100% (*n* = 6), and allantoic injection 15% (*n* = 13). There were no apparent issues or differences in success rates arising from the differing volume loads to the yolk sac.

### 3.3. Tumor Histology and Hypoxia

**Figure 2:**
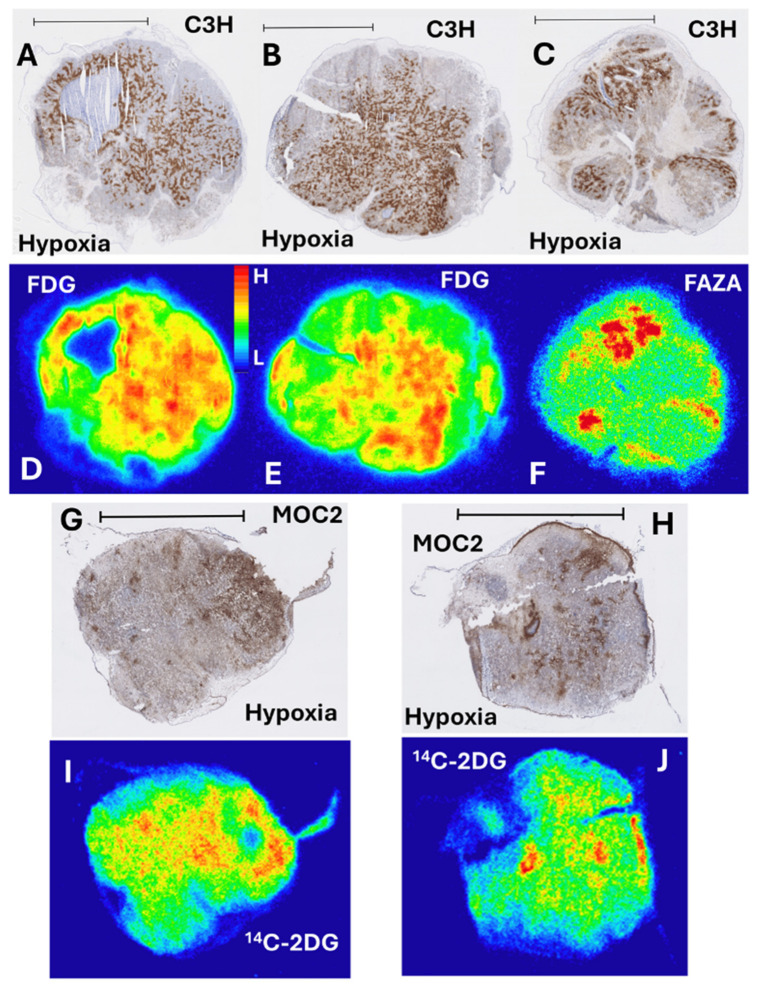
Examples of tissue hypoxia (pimonidazole) and complementary FDG, ^14^C-2DG and FAZA autoradiograms in 2 tumor models. The figure shows examples of pimonidazole stainings (hypoxia) (**A**–**C**) and corresponding autoradiograms (**D**–**F**) for 3 C3H mammary carcinomas administered FDG or FAZA and 2 MOC2 squamous cell carcinomas (**G**–**J**) administered ^14^C-2DG. Of note, there is a clear spatial overlap between areas of low oxygenation and high glucose use in the C3H mammary carcinomas (compare **A** and **B** with **D** and **E**). As expected, the PET hypoxia tracer FAZA (**F**) and pimonidazole-adducts (**C**) distributed similarly. The scale bar equals 5 mm. Autoradiograms are adjusted to optimize image contrast and visual appearance, which does not affect analyses. Red is highest (H) and blue is lowest (L) tracer uptake level.

Examples of hypoxia and underlying histology (pimonidazole counterstained with hematoxylin) and ^14^C-2DG/FDG/FAZA autoradiography for C3H mammary carcinomas and MOC2 oral squamous carcinomas are presented in Figure 2. Tumor necrosis was typically modest. Tracers administered in-yolk-sac were detectable in tumor tissue 3 h post-administration (for further details on tracer uptake and kinetics, see subsequent sections). Tumor hypoxia was widespread, especially in the C3H mammary carcinomas and the hypoxia PET tracer FAZA distributed similarly to pimonidazole. Interestingly, FDG retention was elevated in hypoxic areas in the C3H mammary carcinomas.

### 3.4. PET Scan Results

**Figure 3:**
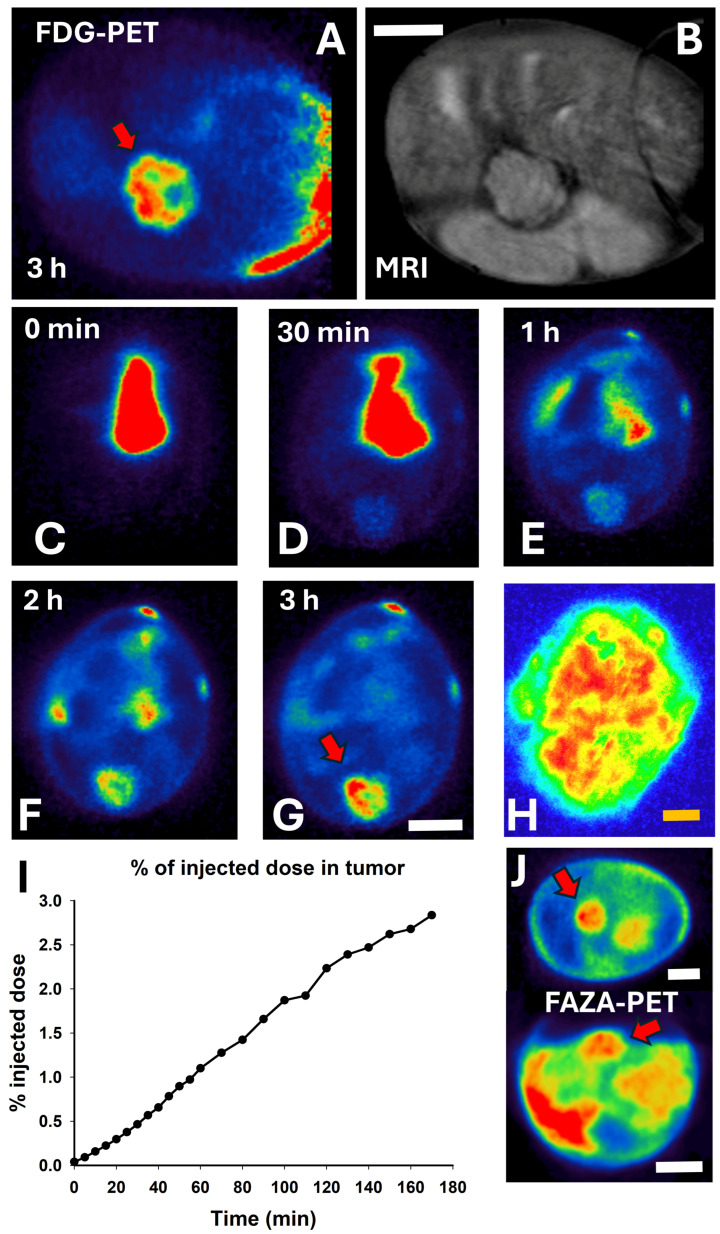
PET scans in tumor-inoculated eggs. (**A**,**B**): Complementary PET and MR images 3 h after in-yolk-sac administration of FDG. (**C**–**G**): Change in tracer distribution over time in the embryo and tumor. (**H**): An autoradiogram for a tissue section obtained from the same tumor. (**I**): A time–activity curve for the depicted tumor. (**J**): FAZA-PET image obtained 3 h after tracer administration in a different tumor. Red arrows show the tumors embedded in the CAM. The white scalebars (PET images) have a length of 10 mm, whereas the orange scalebar (autoradiogram) has a length of 1 mm.

Delivery via in-yolk-sac injection and dripping after scoring consistently resulted in pronounced tumor tracer accumulation. Examples of tracer accumulation in dynamic PET, MRI and static PET analyses are shown in Figure 3.

### 3.5. FDG and ^14^C-2DG Overlap

**Figure 4:**
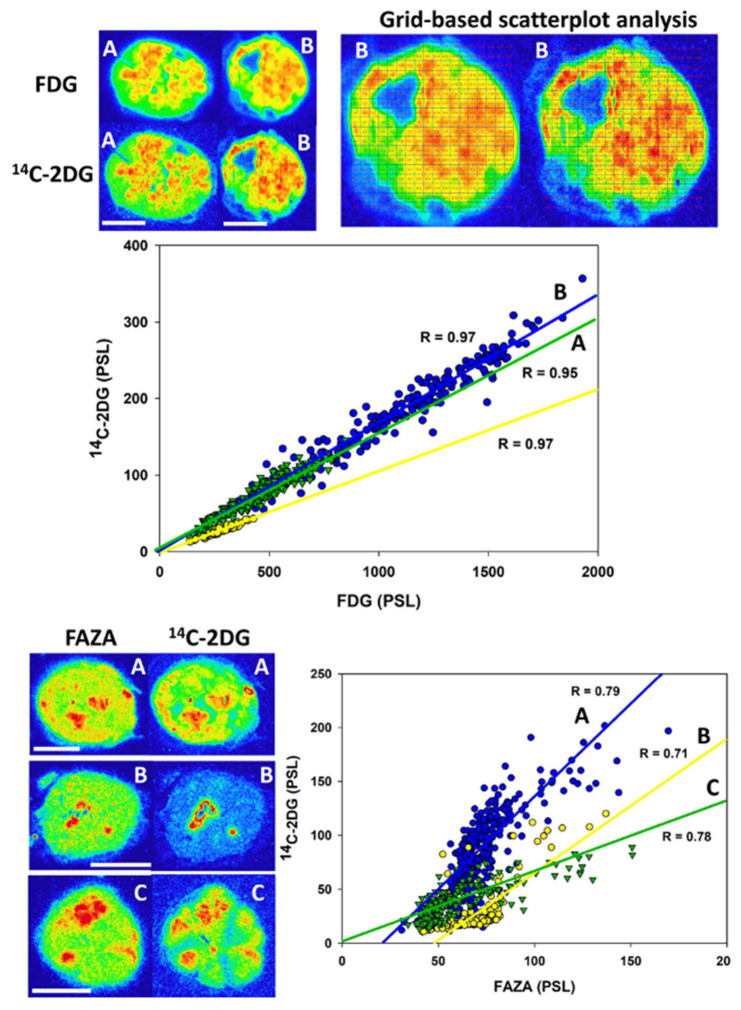
^14^C-2DG is a valid substitute for FDG in autoradiographic studies and ^14^C-2DG uptake is consistently elevated in FAZA-positive areas. Visual inspection of complementary autoradiograms reveals a remarkable similarity in intratumoral distribution patterns of the two glucose tracers. The visual interpretation was confirmed by scatterplot analysis in three tumors (autoradiograms are only shown for 2), where autoradiograms were covered by a grid with a cell size of 0.5 × 0.5 mm (shown in the second panel) followed by regression analysis and calculation of Pearson regression coefficients. Next, a comparison between ^14^C-2DG and the PET hypoxia tracer FAZA revealed an unexpectedly strong similarity between their intratumoral distribution patterns. For clarity, autoradiograms and corresponding regression lines are marked with capital letters. The white bars in the autoradiogram have a length of 5 mm.

To investigate whether ^14^C-2DG is a reliable mimic for the commonly used PET tracer FDG, FDG and ^14^C-2DG were co-injected, and their spatial overlap was evaluated in three tumors. Results shown in Figure 4 illustrate that the two tracers of glucose metabolism distribute identically with R^2^-values close to one. Furthermore, the dynamic range for the two glucose tracers was nearly identical when calculated for each of the three tumors—(FDG vs. ^14^C-2DG): 4.17 vs. 4.56, 2.23 vs. 2.14 and 4.37 vs. 4.25—showing that FDG and ^14^C-2DG can be used interchangeably in autoradiographic studies in the C3H mammary carcinoma CAM model.

### 3.6. FAZA and ^14^C-2DG Analysis

Having demonstrated that FDG and ^14^C-2DG behaved similarly, we next analyzed the overlap of the hypoxia-specific PET tracer FAZA and ^14^C-2DG to answer whether FDG-PET may provide similar information in the C3H tumor model to the use of truly hypoxia-selective tracers. Figure 4 shows data from three different tumors. Quantitative scatterplot analysis revealed a substantial positive correlation between the two tracers.

### 3.7. FDG and ^14^C-Acetate

**Figure 5:**
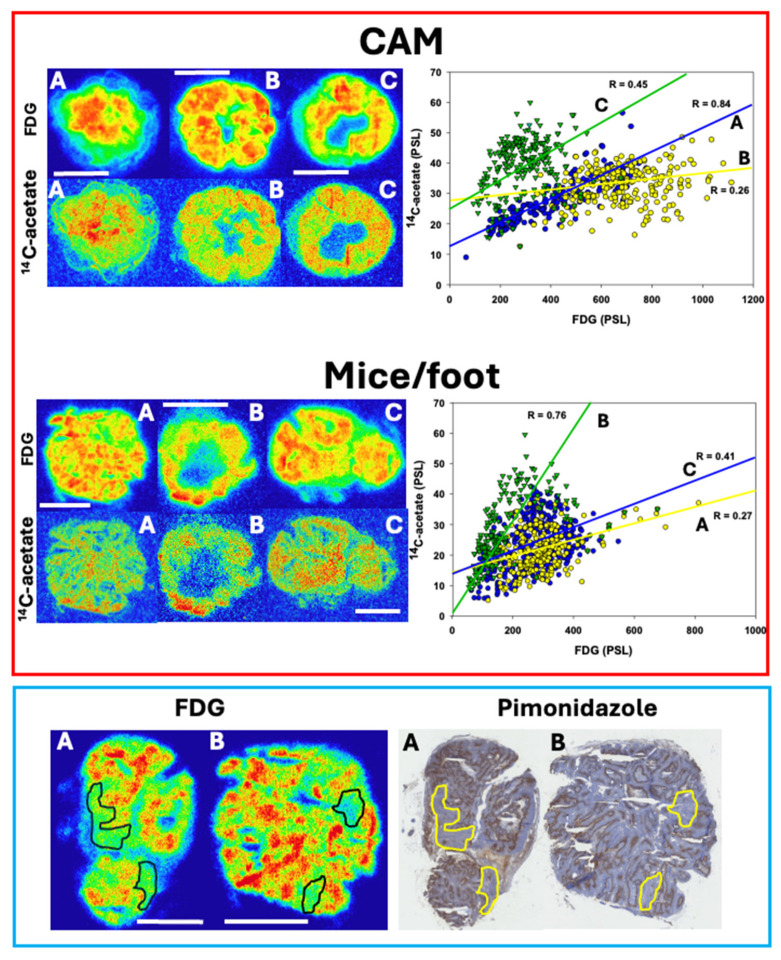
FDG/^14^C-acetate dual-tracer autoradiography in CAM and mice (red box) and FDG-autoradiography/pimonidazole in mouse tumors (blue box). Tumors were analyzed as outlined in Figure 4. Scatterplots revealed a significant, yet highly variable, spatial overlap between the retention of FDG and ^14^C-acetate in both in ovo and in vivo tumors. Due to widespread hypoxia (pimonidazole), the extent of hypoxia-driven FDG is difficult to assess, but areas with little hypoxia (yellow ROIs) displayed relatively low levels of FDG retention (black ROIs), which corroborates well with the FAZA/^14^C-2DG analyses in CAM-grown tumors (Figure 4). For clarity, autoradiograms and corresponding regression lines and pimonidazole stainings are marked with capital letters. The white bars in the autoradiograms have a length of 5 mm.

Furthermore, we investigated the overlap between FDG and ^14^C-acetate, since acetate may serve as an important fuel in energetically stressed cells. Results for three tumors are presented in Figure 5 and show a variable spatial correlation between the two tracers.

### 3.8. Comparison of In Ovo and In Vivo Tumors

Finally, to compare the microenvironment and metabolism between *in ovo* and in vivo tumors, 5 C3H mammary carcinoma tumor-bearing CDF1 mice were administered with FDG, ^14^C-acetate and pimonidazole when tumors reached a size of 0.42 ± 0.05 g. In accordance with the CAM tumor results, there was a variable spatial overlap between FDG and ^14^C-acetate. Due to the widespread pimonidazole staining, a systematic comparison between FDG and hypoxia was not meaningful, but viable areas with little pimonidazole signal were characterized by relatively low FDG signal, which corroborates with the FAZA/^14^C-2DG results for CAM tumors. The results are summarized in Figure 5.

## 4. Discussion

Our findings suggest that the CAM model holds potential for preclinical evaluation of novel radioactive tracers and for characterizing tumor biology using established tracers. Most CAM tumor studies have initiated tumor growth directly from tumor cell cultures resuspended in Matrigel. However, this approach presents limitations, primarily due to the short experimental window inherent to the CAM model. Firstly, the final achievable tumor volume is often too small to assess tumor heterogeneity from PET scans. Typically, tumor volumes are highly variable, depending on days of growth, tumor type and use of Matrigel [19,20,21]. Secondly, although Matrigel accelerates growth and increases take rate, Matrigel hardens after administration, making it difficult to distinguish tumor tissue from Matrigel without histological confirmation [22,23]. Accordingly, a key goal in this study was to develop a robust approach resulting in generally larger tumors. Therefore, we utilized freshly prepared tumor fragments derived from fast-growing (~2 weeks to reach 1000 mm^3^) solid murine C3H flank tumors. This approach circumvents the initial lengthy process of establishing a suitable tumor extracellular matrix, which is essential for tumor expansion. As a result, we achieved substantially larger tumors than most studies have previously reported. Despite fast growth, the tumors did not develop massive central necrosis, which is advantageous in imaging studies. The C3H tumor model was used for the core project, including the testing of various tracer administration routes and tracer combinations, but to confirm the general usefulness of fast-growing murine tumor models, we also tested the MOC2 model in a small cohort of eggs. Again, the resulting tumor size was considerably larger than typically reported in the literature and necrosis was largely absent (Figure 2).

The CAM model is immunodeficient and cannot sustain tumor growth beyond the apoptotic regression of the chorioallantoic membrane near hatching. Furthermore, CAM model preparation involves opening the eggs and purposefully introducing foreign material onto compromised parts of the membrane, which may further impact embryo viability due to contamination. Despite these challenges, we successfully established large tumors with robust CAM model survival, allowing us to proceed with tracer delivery experiments.

Simply depositing FDG (in saline) directly on the intact CAM did not result in any appreciable uptake (see Supplementary Materials), suggesting that the membrane is nearly impermeable. We next scored the membrane gently before dripping, which ensured robust tumor tracer uptake. Still, such a procedure may not be ideal, since it is difficult to standardize and may lead to unwanted damage to the membrane. Therefore, we moved on to test whether allantoic injection is a useful alternative to technically challenging IV injections. The primary functions of the allantois are metabolic waste storage, gas exchange, acid-base homeostasis and water/ion reabsorption [24], so our finding of insignificant FDG uptake is not surprising. Regardless, the uptake and metabolism of ^13^C-labeled glucose administered in the allantoic fluid were previously demonstrated in developing chicken eggs [25]. However, in that study, the focus was on the relative metabolic fate of absorbed glucose (measured over days) rather than uptake rates. Indeed, the authors argued for the relevance of co-injecting a tracer for simultaneous assessment of uptake rate per se. A single C3H tumor displayed robust FDG signal which, ironically, may imply an unrealized faulty injection in a non-defined area outside the allantois. Taken together, our results suggest that uptake from the allantois is insufficient for the use of traditional ^18^F-labeled PET tracers like FDG. Whether the allantois may still be a useful alternative when using long-lived ^14^C labeled tracers remains unanswered. In-yolk-sac injection was chosen next, as it showed consistently high success rates. The entire administered volume is also available for uptake by the organism using an in-yolk-sac injection. In contrast, the dripping after scoring method limits absorption to scored parts of the membrane, with no appreciable uptake taking place elsewhere. This finding was confirmed by PET scans of FDG placed directly onto the intact CAM (see Supplementary Material Figure S1). We demonstrated that an in-yolk-sac injection of various tracers and pimonidazole results in effective distribution to the tumor. CAM studies using IV FDG administration have shown rapid tumor uptake and excellent image contrast 1 h post-injection [5]. Not unexpectedly, in-yolk-sac injection slowed systemic tracer distribution kinetics profoundly, with rather low tumor uptake and image contrast 1 h following tracer administration (Figure 3). To overcome this obstacle, we extended the tracer distribution time to 3 h and used a relatively high dose of 35 MBq to compensate for radioactive decay, which ensured robust tumor uptake and good intertissue contrast (Figure 3). Furthermore, besides being technically undemanding, the in-yolk-sac injection permits administration of bolus volumes up to 0.75 mL in seconds, showcasing easy and rapid delivery of large volumes. In conclusion, in-yolk-sac administration offers a robust alternative to intravenous injection in the CAM model, given its high success rate and relative procedural simplicity. The exact success rate of IV injections is rarely reported, but in one study the success rate was 75%, while we attained 91% success using in-yolk-sac administration [6].

Having established the reliability of in-yolk-sac delivery, we evaluated the CAM model’s capacity to address questions relevant to the imaging community. Clinically, the glucose analog FDG is commonly used in PET scans for oncological applications, whereas its long-lived analog ^14^C-2DG is often employed preclinically, since it can be stored and does not require an onsite cyclotron. Although both tracers share an identical intracellular trapping mechanism (glucose-transporter-mediated uptake followed by phosphorylation to a membrane-impermeable form), their chemical structures differ. A similar systemic and intra-tumoral distribution is, therefore, not trivial. Accordingly, we tested the spatial distribution overlap using dual-tracer autoradiography. As illustrated in Figure 4, the two tracers were distributed identically. Furthermore, the dynamic ranges for the two glucose analogs were nearly identical. Therefore, ^14^C-2DG was deemed a suitable substitute for FDG in subsequent dual-tracer autoradiographic studies. We then investigated whether FDG-PET could be a suitable alternative to hypoxia-PET. Tumor hypoxia is widespread and linked to poor outcome, partly due to distinct radioresistance of viable hypoxic cells [8]. Systemic treatment with radiosensitizers (e.g., nimorazole) or dose escalation to hypoxic tumors or hypoxic tumor regions (so-called dose-painting) may overcome this radioresistance [26]. While experimental hypoxia-selective tracers like FMISO, HX4, or FAZA can accumulate in viable hypoxic (pO_2_ < 10 mmHg) cells, thereby offering prognostic value and an incentive for treatment intensification, none of them are implemented in routine clinical practice. It is well-known that hypoxia promotes glucose uptake and O_2_-independent glycolytic ATP production in animal cells, suggesting a possible role of FDG-PET in hypoxia imaging [27]. Since dose-painting based on regional metabolic/proliferative activity, as quantified from FDG-PET, has also received considerable interest [28,29], understanding similarities and differences between FDG-PET and hypoxia-PET is of significant interest for the radiotherapy community. Glucose consumption varies with metabolic needs (e.g., cell type and proliferation), and tumor cells’ inherent dependency on glycolytic ATP production, even under well-oxygenated conditions (the so-called Warburg effect or aerobic glycolysis) may dampen the oxygen-driven component of increased glucose uptake [30]. Furthermore, insufficient glucose/FDG availability in hypoxic areas or adaptive energy-saving metabolic rate depression (cellular dormancy) may further weaken any regional or global correlation between local FDG uptake and oxygenation. Given the previous discussion, it is unsurprising that clinical studies have demonstrated poor correlations between traditional FDG-PET SUV metrics (e.g., mean/max) and global accumulation of hypoxia-selective tracers, suggesting that such simple metrics are unreliable for identification of hypoxic tumors. Nonetheless, if hypoxia *per se* is a prominent driver of glucose consumption, FDG-PET images may still have a role for mapping of hypoxic foci, which could guide dose painting strategies. Several patient studies have compared FDG- and hypoxia-PET maps using voxel-by-voxel scatterplot analysis. Findings have been highly inconsistent across tumor types, but also in comparable patient groups, suggesting methodological problems like co-registration issues or true biological changes related to the obligatory time separation (at least one day) between the two scans, which may obscure true similarities [8]. Dual-tracer autoradiography using a hypoxia-selective tracer like FAZA with ^14^C-2DG can fully circumvent these technical limitations by showing glucose metabolism and hypoxia distribution within a single tumor section at a high resolution. Using this approach, we demonstrated that ^14^C-2DG is consistently elevated in areas with high FAZA uptake, suggesting that FDG-PET, in the C3H model, may be a suitable substitute for hypoxia-selective PET for mapping intratumoral hypoxia (Figure 4). Interestingly, a similar conclusion was reached using the same methodology in a selection of murine or human orthotopic and subcutaneous tumor models established in mice [31]. Additionally, the MOC2 tumor presented with areas of overlap and areas of mismatch when comparing ^14^C-2DG and pimonidazole (Figure 2), suggesting tumor-to-tumor model differences. In another study [32], we showed that in vivo tracer distribution patterns could be attributed to inherent metabolic energetic phenotypes with good spatial correlation between high FDG uptake and hypoxia (pimonidazole) in tumors grown from cells with an atypical non-Warburg energetic phenotype. Unfortunately, despite several attempts, we have not been able to establish the C3H model as a cell line. Further studies are warranted to assess the linkage between in vitro inherent energetic phenotypes and the in vivo hypoxia-driven component of FDG retention, and how such tumors can be identified prior to treatment (e.g., biopsy-based assessment of genetic differences). Our dual-tracer methodology is ideal for such studies since it overcomes resolution problems and movement and time-separation-related artifacts.

Acetate is hypothesized, largely based on cell culture studies, to serve as an alternative fuel source for tumor cells under energetic stress, including low oxygen and/or low glucose conditions [10,14]. It is converted to acetyl-CoA by the enzyme ACSS2, which may be essential for tumor growth under nutrient-limiting conditions, presenting a potential therapeutic target to overcome the negative influence of treatment-resistant hypoxic cells. However, the intratumoral distribution of acetate remains poorly characterized, and given that plasma acetate concentrations are much lower than those of glucose, it remains unknown whether acetate is a quantitatively important fuel and metabolite in glucose- and/or oxygen-deprived tumor regions. Dual-tracer autoradiography enables high-resolution spatial mapping of acetate metabolism and facilitates direct comparison between retention of other tracers such as FDG and immunodetectable tracers (e.g., pimonidazole), while also providing histological features. Therefore, we tested the usefulness of the CAM model and dual-tracer autoradiography for providing in vivo data that may lend support to the intriguing idea that treatment-resistant cells may be susceptible to the targeting of acetate uptake/metabolism. Our results suggested a variable correlation between FDG and acetate uptake (Figure 5) but did not support elevated uptake of acetate in areas with low glucose uptake in the C3H model. Dual-tracer autoradiographic analyses of ^14^C-acetate and FDG and ^14^C-acetate and FAZA (or pimonidazole) in a broad selection of tumor models would clearly be of interest for the imaging community to clarify whether acetate is a quantitatively important, and targetable, fuel in metabolically stressed cells in vivo, and the CAM model offers a promising, relatively high-throughput platform for such experiments.

Finally, we included results from five C3H mammary carcinomas grown SC in mice. The results were largely in agreement with CAM C3H tumors, with a variable spatial overlap between FDG and ^14^C-acetate (Figure 5). Interestingly, a qualitative visual comparison between pimonidazole-adduct formation (hypoxia) and FDG revealed some correlation, which resembled the results obtained in CAM tumors for FAZA and ^14^C-2DG. Taken together, CAM-grown tumors recapitulate the tracer distribution patterns observed in traditional SC mouse tumors.

## 5. Conclusions

The chicken CAM model employing a fragment grafting approach demonstrates high grafting efficiency and robust embryo survival, and enables the growth of generally larger tumors than previously reported. To our knowledge, this study is the first to present advanced dual-tracer autoradiography data derived from tumor material grown using the CAM model. Furthermore, an in-yolk-sac injection method has been investigated for the first time as a reliable and user-friendly alternative administration method for successfully delivering substantial volumes to the model’s circulatory system. Collectively, these methodological refinements highlight the CAM model’s value as a cost-effective and time-efficient platform for investigating tumor metabolism with a lower ethical burden than traditional murine studies. Nevertheless, further investigation is warranted to determine the extent to which the findings in the avian system translate to traditional mammalian models.

## Data Availability

The raw data supporting the conclusions of this article can be made available by the authors upon request.

## Supplementary Materials

The following supporting information can be downloaded at: https://www.mdpi.com/article/10.3390/biomedicines14071515/s1, Figure S1: PET scan of direct FDG dripping onto intact CAM; Table S1: Growth statistics.

## Abbreviations

The following abbreviations are used in this manuscript:

^14^C-2DG	^14^C-2-deoxyglucose
CAM	Chorioallantoic membrane
CI	Confidence interval
EDD	Embryonic development day
FAZA	^18^F-Fluoroazomycin-arabinoside
FDG	^18^F-fluorodeoxyglucose
PCC	Pearson correlation coefficients
SC	subcutaneously
