# Supplementary material for: Dual-Tracer Autoradiography and Positron Emission Tomography (PET) Scans Using In-Yolk-Sac Tracer Delivery in the Chicken Chorioallantoic Membrane (CAM) Tumor Model
Source: Biomedicines. 2026 Jul 6;14(7):1515. doi: 10.3390/biomedicines14071515 (PMC13405463; doi:10.3390/biomedicines14071515)
Supplement: Supplementary file 1 [file biomedicines-14-01515-s001.zip › biomedicines-4332237-supplementary.pdf]

Supplementary Figure S1 - FDG-administration directly onto the CAM

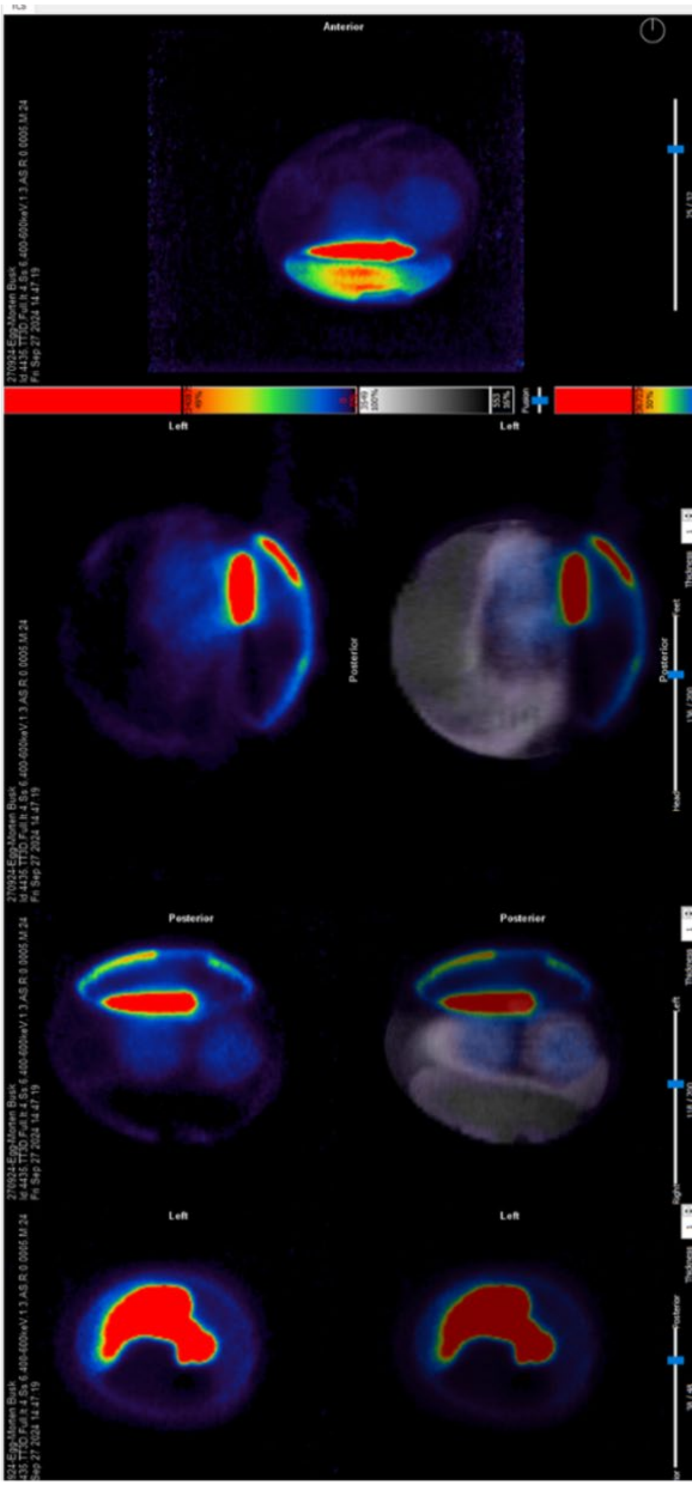


**Supplementary Figure S1.** Pilot positron emission tomography (PET) scan and MRI showing negligible uptake of FDG through the CAM via direct dripping without scoring. Left column shows PET scan and right column shows PET + MRI overlay with no uptake to the fetus itself through the CAM.

**Supplementary Table S1.** Growth statistics for each tumor type.

| Tumor Type | Mean graft weight (g) [95% CI] | Mean Tumor Weight (g) [95% CI] | Mean Tumor Growth (%) [95% CI] | Take Rate (%) [95% CI] |
|---|---|---|---|---|
| MOC2 oral squamous cell carcinoma | 0.038 [0.034–0.042] | 0.189 [0.087–0.291] | 391.5 [176.1–870.4] | 66.7 [30.0–90.3] |
| C3H mammary carcinoma | 0.049 [0.046–0.052] | 0.319 [0.276–0.367] | 555.4 [451.7–683.0] | 81.0 [70.1–88.5] |

Supplementary Table S1 shows the growth of 68 C3H mammary carcinomas and 4 MOC2 oral squamous cell carcinomas. Takers were defined as tumors with >100% growth compared to initial graft weight.
